# Supplementary material for: Splice-Junction-Based Mapping of Alternative Isoforms in the Human Proteome
Source: Cell Rep. Author manuscript; Available in PMC 2020 Jan 15. (PMC6961840; doi:10.1016/j.celrep.2019.11.026)
Supplement: 2 [file NIHMS1546469-supplement-2.zip › DF1/PXD010154/Thyroid/Thyroid_38_TG_GASQILLASICWVSANIFEYQVDAQPLRPCELQR.pdf]

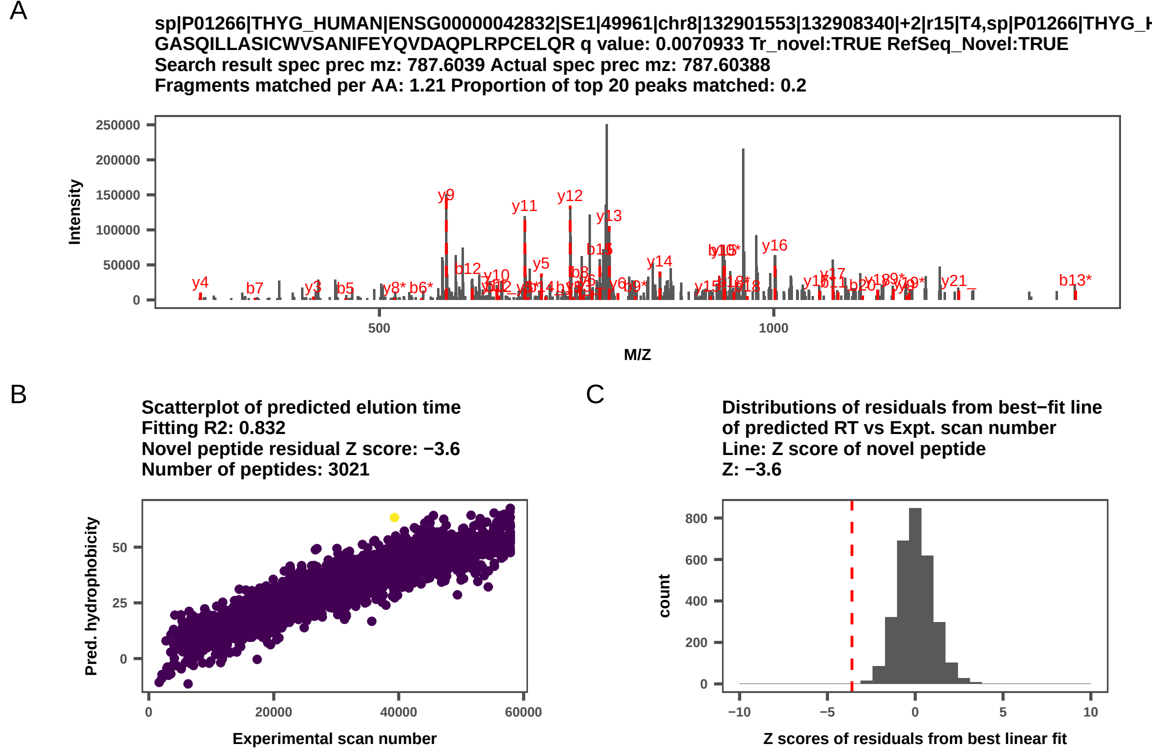
A
sp|P01266|THYG_HUMAN|ENSG00000042832|SE1|49961|chr8|132901553|132908340|+2|r15|T4,sp|P01266|THYG_H
GASQILLASICWVSANIFEYQVDAQPLRPCELQR q value: 0.0070933 Tr_novel:TRUE RefSeq_Novel:TRUE
Search result spec prec mz: 787.6039 Actual spec prec mz: 787.60388
Fragments matched per AA: 1.21 Proportion of top 20 peaks matched: 0.2
Intensity
250000
200000
150000
100000
50000
0
y9
y11
y12
y13
y16
y14
y5
b12
y10
y4
b7
y3
b5
y8*
b6*
b13*
y21
500
1000
M/Z
B
Scatterplot of predicted elution time
Fitting R2: 0.832
Novel peptide residual Z score: −3.6
Number of peptides: 3021
Pred. hydrophobicity
50
25
0
0
20000
40000
60000
Experimental scan number
C
Distributions of residuals from best−fit line
of predicted RT vs Expt. scan number
Line: Z score of novel peptide
Z: −3.6
count
800
600
400
200
0
−10
−5
0
5
10
Z scores of residuals from best linear fit
